# Despite Potential Risks African Elephants Do Not Always Avoid Mountaineering

**DOI:** 10.1002/ece3.71753

**Published:** 2025-07-07

**Authors:** Justine M. Teixeira, Rickert van der Westhuizen, Adrian M. Shrader

**Affiliations:** ^1^ Department of Zoology and Entomology University of Pretoria Pretoria South Africa; ^2^ Ezemvelo KZN Wildlife, Cascades Pietermaritzburg South Africa

**Keywords:** accident landscape, landscape use, *Loxodonta africana*, slope use

## Abstract

As herbivores forage, they move across a wide range of topographical features. Yet, they tend to avoid terrain such as steep slopes where energetic costs of movement are high and there is a greater risk of falls and tumbles. Recent studies suggest that African elephants ( *Loxodonta africana* ) avoid steep slopes (e.g., > 15°). However, in reserves with undulating topography, elephants may have to use steep slopes to obtain food, especially when availability is limited in more gradual areas. To explore this, we investigated slope use by elephants in the Ithala Game Reserve, South Africa, where the topography varies greatly and ranges between 400 to 1400 masl. Using 8.5 years of positional data, we examined how slope use varied between herd types (14 breeding herds and 13 males), habitat type and season (wet vs. dry). Elephants were found primarily on slopes < 30° (i.e., 95% of the locations), while 67% of the locations were on slopes < 15°, and 52% on slopes < 10°. Breeding herds used steeper slopes (mean = 12.6° ± 0.08 SE) than bulls (mean = 12.0° ± 0.8 SE). In addition, habitat influenced slope use, with the steepest slopes used in woodlands and the most gradual used in built‐up areas and grasslands. However, these slope use differences were very small (i.e., 0.6° to 9.7°) and thus unlikely to be biologically meaningful. Rather, the ability to detect these slight differences was likely an artefact of our large sample size (*N* = 23,837 locations). Moreover, slope use did not vary between the wet and dry seasons. Nevertheless, 5% of all the elephant locations occurred on very steep slopes (i.e., > 30°) and 33% were on slopes > 15°, indicating that although they may prefer flat terrain, when required, elephants will mountaineer.

## Graphical abstract

Recent studies have suggested that African elephants ( *Loxodonta africana* ) avoid steep slopes (e.g., > 15°) due to energetic costs of movement and the greater risk of falls and tumbles. Yet, using 8.5 years of positional data, we found that elephants in the Ithala Game Reserve, South Africa primarily used slopes < 30° (95% of the locations), while 67% of the locations were on slopes < 15°, and 52% on slopes < 10°. These results indicate that despite elephants apparently preferring flat terrain, when required, they will mountaineer.

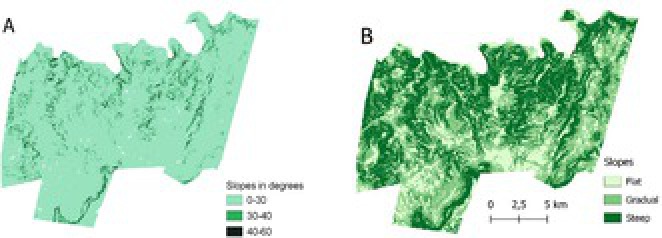

## 1 Introduction

Landscape use by herbivores is driven by several variables, including the availability of resources, predation risk and the topography of the landscape (Shrader et al. 2008; Mariotti et al. 2020). As they move across landscapes, herbivores must weigh up the costs and benefits of each of these factors. For example, while foraging, herbivores gain energy and nutrients from the plants they consume. However, they experience energetic costs as they search for food (Pretorius et al. 2012). As the availability of resources varies with season, habitat, and topography (Kim and Eltahir 2004; Shrader et al. 2012; Lakshimaryanan et al. 2016; Soto‐Shoender et al. 2018), all three can influence landscape use by herbivores.

Undulating terrain provides challenges to herbivores with regards to energy costs, with greater effort required to move up steep slopes (Wall et al. 2006). Coupled with this, areas such as steep slopes, and loose substrate can increase the difficulty of movement and thus increase the risk of falls, slips and tumbles (Wheatley et al. 2021). As such, these factors help define an animal's ‘accident landscape’ with areas where accidents are more and less likely to happen. Yet, these accident landscapes vary depending on the species size, vulnerability, and ability to move across different topography (Wheatley et al. 2021).

Due to their large size, topography such as elevation and slope, play key roles in defining landscape use of megaherbivores such as African elephants ( *Loxodonta africana* ) (Wall et al. 2006; Kimuyu et al. 2021). Elephants move in herds, with breeding herds consisting of females and their dependent offspring, while males can be solitary or move in smaller, weakly associated bachelor herds (Shannon et al. 2006). As the individuals comprising these herds differ, the herds likely perceive the landscape differently with regard to accident risk (Kathreen and Peter 2005). For example, larger males (ca. 6000 kg vs. ca. 4000 kg females; Skinner and Chimimba 2005) may experience a greater risk of injury from falling, while the young within breeding herds may have less experience walking along difficult terrain and thus be more susceptible to accidents.

Elephants have low metabolic rates and as a result, movement incurs relatively high costs, where climbing upward can expend 2500% of the energy required to move across a flat landscape (Wall et al. 2006). It is expected, therefore, that megaherbivores would avoid resource patches on steep slopes or in depressions (Kimuyu et al. 2021) owing to the transport costs associated with moving through these types of terrain (Gallagher et al. 2017). In addition, megaherbivores might aim to optimise the trade‐off between the benefits associated with foraging and the costs of energy expenditure, and risk of injury (Wheatley et al. 2021). However, perceptions of accident landscapes may vary seasonally in response to changes in the distribution of food quality and availability (Hutchings et al. 2000).

During the wet season, elephants feed predominantly on grass (Codron et al. 2011; Kos et al. 2012), but then shift and forage more on woody plant material during the dry season (Codron et al. 2011). However, in the Ithala Game Reserve (IGR), South Africa, the elephants tend to feed primarily on woody vegetation and consume very little grass (Shrader et al. 2012). Nevertheless, to make seasonal adjustments in their diet, elephants typically shift and forage in different habitats and portions of the landscape (Shrader et al. 2012), some of which they may have previously avoided (Mariotti et al. 2020). As the dry season progresses, food availability declines due to deciduous plants senescing and losing their leaves (Abraham et al. 2019), while availability decreases due to utilisation by a range of herbivore species (Ferry et al. 2016). In response, megaherbivores such as elephants may need to utilise more dangerous portions of their accident landscape to obtain adequate food (e.g., feed on steeper slopes). This pattern was recorded for white rhinos in the Hluhluwe‐iMfolozi Park, South Africa, where during a drought, white rhinos were observed to feed on previously avoided steep slopes to obtain the only remaining grass (Owen‐Smith 1988).

Wall et al. (2006) found that elephants avoided the use of a prominent, isolated hill despite the abundant resources available on it. They suggested that the reason for this was due to the high energetic cost that elephants would experience if they walked up the steep slopes. However, not all elephants live in areas that are predominantly flat with very few hills. In fact, a majority of the world's protected areas are biassed towards remote areas with high elevations and steep slopes (Joppa and Pfaff 2009). Thus, elephants living in these sorts of protected areas likely have to forage across a range of topographical features and slopes. For example, the IGR comprises undulating terrain that ranges from ca. 400 to 1400 m asl and is home to 265 elephants. Within the reserve, these elephants do not have access to large expanses of flat terrain. Thus, understanding how the elephants utilise the undulating landscape provides valuable insight into the full range of slopes that elephants may use. This then helps managers in IGR and other protected areas better quantify the full extent of the landscape and resources available to elephants, and therefore the spatial distribution of impacts that they might have. As a result, our study addresses some key questions: (1) What is the maximum slope that elephants are willing to use? (2) Does slope use differ with habitat? (3) How does landscape use vary both seasonally (i.e., wet vs. dry), and (4) Are there differences in the use of accident landscapes between breeding herds and bachelor herds?

Huang et al. (2022) and Roever et al. (2012) found that African elephants tend not to utilise slopes steeper than 3° or 4°, while Asian elephants ( *Elephas maximus* ) will use slopes up to 10° (Wilson et al. 2021). However, Ngama et al. (2019) noted that African forest elephants preferred slopes with mid‐range steepness, 10%–25%, which is equivalent to 5°–15°, as opposed to steep slopes (> 25% i.e., > 15°). Understanding that the elephants in the IGR have limited access to flat areas, the objectives of our study were to determine the full extent of the slope use by the elephants and the environmental factors that drive that use. We predicted that they would utilise slopes between 0° and 15°. Yet, as food availability and quality declined during the dry season, we predicted that the elephants would increase their use of steep slopes to access available resources. Moreover, as the overall availability and quality of food tend to be lower during dry years (i.e., below average rainfall), we expected elephants to use steeper slopes during dry years compared to average rainfall years. Finally, due to the greater risk of accidents to younger individuals, we expected that breeding herds would not use as steep of slopes as bulls. However, alternatively, as bulls are larger than cows and thus the risk of injury from falling is likely greater for bulls, it is possible that bulls would utilise less‐steep slopes compared to breeding herds. Ultimately, understanding how elephants utilise their landscapes provides an estimate of the proportion of a protected area that is truly accessible to elephants and thus helps inform management practices for the conservation of both elephant populations and habitats within protected areas (Scheiter and Higgins 2012).

## 2 Materials and Methods

### 2.1 Study Site

We conducted our study in the 291 km^2^ IGR, Kwa‐Zulu Natal, South Africa (27°45′ S, 31°37′ E). The reserve is fenced along its eastern, western, and southern boundaries, with the unfenced Pongola River acting as the northern boundary. Despite water flowing in the river year‐round, elephants do periodically make short excursions across the river and forage outside the reserve (Ward et al. 2017, Ezemvelo unpublished data). However, these excursions only tend to last 1 or 2 days. During our study, the elephants utilised the entire park throughout the year (Ezemzelo KZNWildlife unpublished data), and did not display the seasonal utilisation pattern recorded through 2011 of preferring the east of the reserve during the wet season and the west in the dry season (see Ward et al. 2017).

To determine seasonal slope use by the 265 elephants in the IGR, we used GPS location data collected from satellite collars fitted to 27 individuals (14 females, 13 males). Each individual was from a separate herd, with the females in breeding herds that included young, while the males were either in bachelor herds or solitary. Data collection ran from November 2014 to April 2023 (ca. 8.5 years). During 2014 to 2019, GPS positions were recorded every 3 h, while during 2020 to 2023 positions were recorded every hour.

We divided the location data into seasons (wet or dry) based on monthly rainfall. Dry season months (April–September) received ≤ 35 mm of rain, and wet season months (October to March) received ≥ 60 mm of rain. In addition, we explored whether the locations were recorded in above average, average or below average rainfall years. We did this by determining whether the annual rainfall exceeded, fell within or fell below one standard deviation of the mean annual rainfall since 1972 (i.e., 743 mm). During the study, there were only 2 years with below average rainfall (i.e., 2014, 2015), while the remaining years received average rainfall. Thus, we limited our analysis to these two types of rainfall years. To determine which habitat the elephant locations were in, we overlaid the locations on the digitised habitat map for the IRG from Van Rooyen and Van Rooyen (2008), which consisted of 27 different habitat categories. Prior to analysis, however, we grouped these habitat categories into seven functional groups (i.e., built‐up, bushveld, grassland, riparian vegetation, wetlands, wooded grasslands, woodlands) based on vegetation traits (Table S1).

### 2.2 Data Point Selection

During the midday, when temperatures are at their highest, and in the early mornings, elephants tend to spend a number of hours resting (Wyatt and Eltringham 1974; Guy 1976; Kalemera 1987; Shannon et al. 2008). As the collars recorded locations every 1 to 3 h, this would result in multiple locations being recorded for each herd as they rested at the same place. Thus, there would be an overrepresentation of these locations and habitats (primarily woodlands) in the data set. To avoid this temporal autocorrelation of the GPS locations (Boyce et al. 2010; Perotto‐Baldivieso et al. 2012), we selected locations that were separated by a minimum of 13 h (mean = 25 h). This resulted in approximately one location per day per herd. In addition, to ensure that the locations spanned the full 24‐h period and thus captured the diurnal activity patterns of elephants as they foraged, travelled, and rested (Guy 1976; du Plessis et al. 2021), we selected locations for each subsequent day from the next timestamp (i.e., 3 h or 1 h later than the previous day). Together, this process resulted in us including *N* = 23,837 locations in the analyses.

### 2.3 Data Analysis

We imported the elephant locations into QGIS 3.28. Each point was allocated to a habitat type by intersecting the points with the habitat shapefile. Thereafter a 20 m digital elevation model (DEM) was masked to the park boundary and the slope calculated using the ‘slope’ function in the ‘terrain analysis’ package in QGIS. An 8 m buffer was created around each point to account for the GPS accuracy of the satellite collars, while a 10 m buffer around the rivers and roads were created to offset inaccuracies in the shapefile data. The average and standard deviation of the slope were calculated across these buffered areas using the ‘zonal statistics’ package in QGIS.

Elephants frequently move across the landscape by utilising roads and other flat surfaces such as dry riverbanks (Wall et al. 2006, R. van der Westhuizen personal observation). To account for this, if an elephant GPS location fell within the 10 m buffer around a river or road (*N* = 5108 locations), we assumed that it was travelling along that riverbank or road that followed the contour and not straight up the slope. To reflect this, we replaced the slope of the original location with the average slope of the river or road.

**FIGURE 1:**
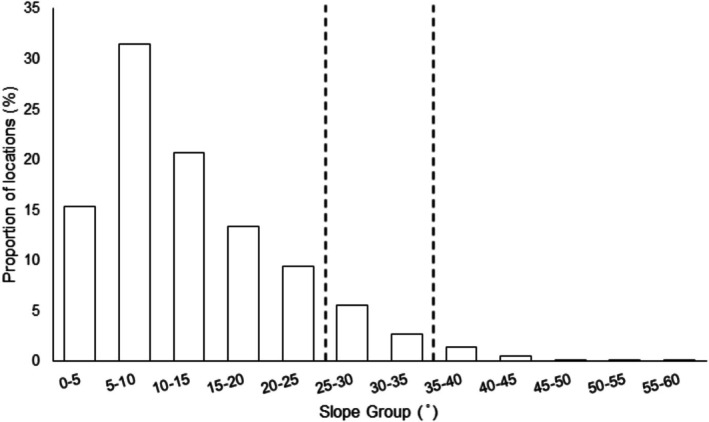
Proportion of the elephant herd locations (breeding herd and males) divided into five‐degree slope use categories.

Based on the distribution of the utilised slopes, each location was categorised into one of three broad slope use categories: < 30°, 30°–40° and > 40° (Figure 1). We determined the natural breaks in the data that defined each of these broad categories by first separating the location data into 5° slope groups (e.g., 0°–5°, 5°–10°, 10°–15°; Figure 1). Then, we calculated a break when the proportion of locations in any subsequent group was less than half of the proportion of locations in the previous group (i.e., 25°–30° = 5.53% to 30°–35° = 2.62%, and 35°–40° = 1.36% to 40°–45° = 0.42%; Figure 1). To determine the extent to which the elephants used each of the broad slope categories, in relation to availability of these categories across the reserve, we calculated the total area of the park (km^2^) as well as the area of each slope category (km^2^). We generated availability using the entire park as in contrast to previous years (see Ward et al. 2017), the elephants did not show seasonal selection for a specific side of the reserve (i.e., West during the dry season, East during the wet season). Rather the elephants utilised the entire park throughout the year.

### 2.4 Statistical Analysis

To determine how elephant slope use varied with the independent variables of herd type, season, year and habitat type, while accounting for the specific elephant herds (i.e., particular collar tag) as a random effect, we ran a generalised mixed linear model with the gamma distribution and a log link function in R (R Core Team 2021). The variables in a gamma distribution model are positively skewed and greater than zero; yet, 117 of the recorded slopes utilised by the elephants were on flat ground with a slope of 0. As these zero values did not reflect a lack of data but rather flat ground, we added 0.001 to all the slopes (*N* = 23,837 slopes), which then allowed us to analyse our data using a gamma distribution.

An important consideration with movement data is spatial autocorrelation in the model residuals. We used the widely accepted Moran's I test statistic (Dormann et al. 2007) to determine the presence and direction of autocorrelation. To account for this, we generated a spatial autocovariate as a predictor (Dormann et al. 2007). The autocovariate was generated as a weighted average of slope values for the 5 nearest neighbours for each location point. The addition of an autocovariate accounted for the autocorrelation caused by endogenous processes (Dormann et al. 2007).

In order to determine the most parsimonious model, we used the automated model selection dredge function, part of the MuMln package in R (Bartoń 2025) on the model that included all predictors, the random effect and the autocovariate. The best model included the main effects of habitat and herd only, while still including the herd ID as a random effect and the autocovariate. Thus, year (below average rainfall, average rainfall) and season (wet, dry) did not influence elephant slope use. However, as changes in season yield biologically relevant changes in landscape use (Shrader et al. 2012; Yoganand and Owen‐Smith 2014; Boyers and Parrini 2024), we opted to include season as a predictor in the model. The best model is that with the lowest AIC and a delta value < 2. Given the closeness in AIC value as well as a delta value still < 1 and a weight of > 1/2 the weight of the best model, the inclusion of season as a predictor did not compromise the model fit (Table S2).

**FIGURE 2:**
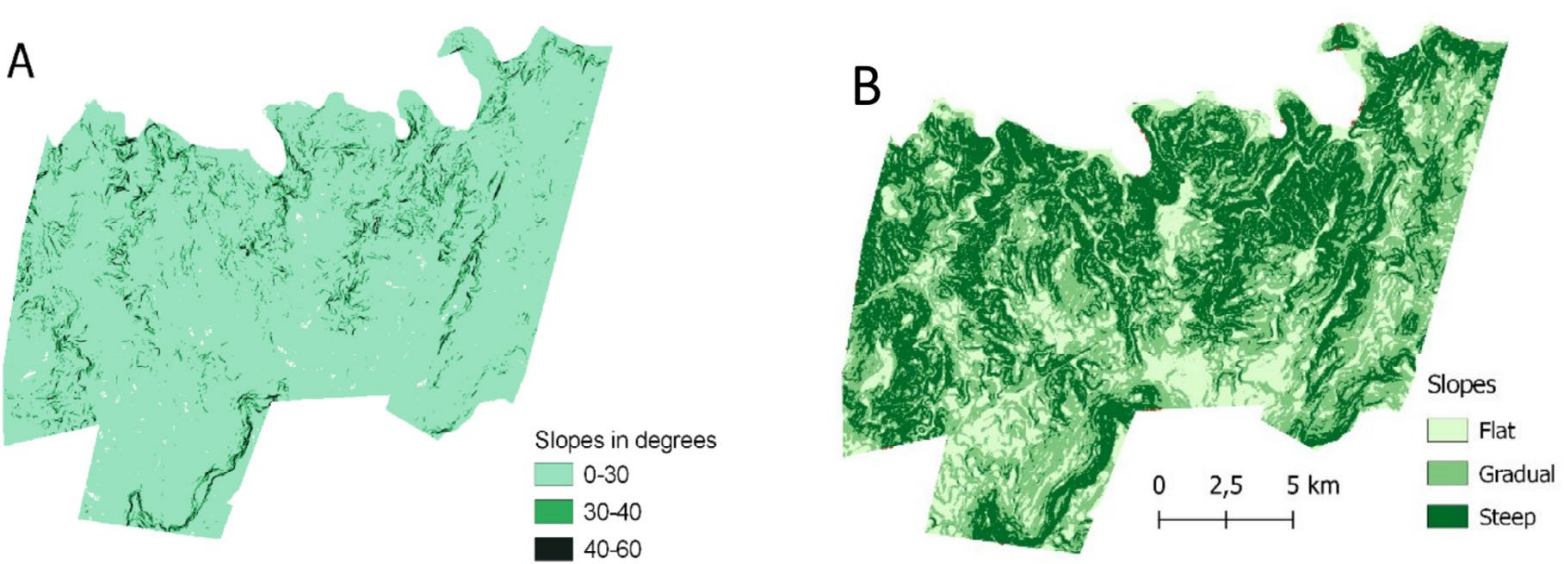
Distribution of the slope categories as defined by (A) the utilisation by the elephants in the current study (i.e., < 30°, 30°–40°, > 40°), and (B) according to the elephant use categories defined by Ngama et al. (2019) where flat slopes are < 5°, gradual/shallow slopes 5°–15°, and steep slopes > 15°.

To determine the extent to which the elephants' slope use differed to what was available across the reserve, we conducted a utilisation‐availability analysis (Byers and Steinhorst 1984) in R with Bonferroni confidence intervals. We did this for the slope classification (i.e., < 30°, 30°–40° and > 40°). In addition, to provide further context of the variability of slopes found within the IGR, we conducted a second utilisation‐availability analysis using the slope categories proposed by Ngama et al. (2019; i.e., flat < 5°, gradual/shallow 5°–15° and steep > 15°), and calculated the availability and distribution of these slope categories within the reserve (Figure 2).

## 3 Results

The IGR covers 291 km^2^, with 264 km^2^ (i.e., 90%) comprised of slopes < 30°, 22 km^2^ (i.e., 8%) comprised of slopes between 30° and 40°, and 5 km^2^ (i.e., 2%) comprised of slopes > 40° (Figure 2). The use of these slopes by the elephants differed from availability (*X* ^2^ = 660.99, df = 2, *p* < 0.0001), with the elephants using slopes < 30° more than expected (95% of the locations), while using slopes between 30° and 40° (4.5% of the locations) and > 40° (0.5% of the locations) less than expected (Table 1). Slopes categorised, according to Ngama et al. (2019) as flat (i.e., < 5°) covered 49 km^2^ (i.e., 17%), gradual/shallow slopes (5°–15°) covered 123 km^2^ (i.e., 42%), and steep slopes (> 15°) covered 119 km^2^ (i.e., 41%) (Figure 2). In contrast to the suggestions of Ngama et al. (2019), the elephants in IGR used gradual slopes significantly more than expected, but the flat and steep slopes less than expected (< 5° = 15% of locations, 5°–15° = 52% of locations, > 15° = 33% of locations; Table 1). The steepest slope that was used by the elephants during our study was 58°, which was recorded for a breeding herd in the wet season, followed by 53° for a male in the dry season.

**TABLE 1:** Utilisation‐availability analysis of the slopes in the Ithala Game Reserve.

Slope category	Expected proportion (Pi_0_)	Utilised proportion (Pi)	Bonferroni interval
< 30°	0.907	0.955	0.952 ≤ *p* ≤ 0.958*
30°–40°	0.076	0.040	0.037 ≤ *p* ≤ 0.043*
> 40°	0.017	0.005	0.004 ≤ *p* ≤ 0.006*
< 5°	0.168	0.153	0.147 ≤ *p* ≤ 0.158*
5°–15°	0.423	0.520	0.528 ≤ *p* ≤ 0.528*
> 15°	0.409	0.327	0.327 ≤ *p* ≤ 0.335*

* Indicates significant difference at 0.05 level.

### 3.1 Habitat

**FIGURE 3:**
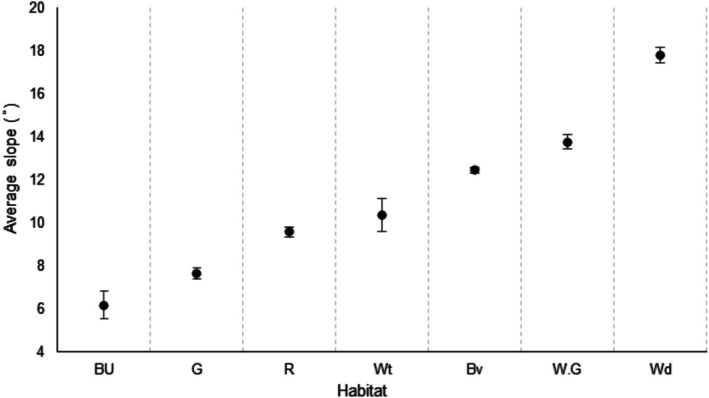
Average slope use with 95% CI of the elephants in each habitat category: Built‐up (Bu), grasslands (G), riparian (R), wetlands (Wt), bushveld (Bv), wooded grasslands (W.G) and woodlands (Wd).

Habitat had a strong influence on the slope use of the elephants (*X* ^2^ = 198.10, df = 6, *p* < 0.0001; Figure 3) with them moving onto steeper slopes in woodlands (mean = 17.5° ± 0.4 SE), wooded grasslands (mean = 13.7° ± 0.3 SE) and bushveld (mean = 12.5° ± 0.3 SE) compared to wetlands (mean = 10.1° ± 0.5 SE), riparian woodlands (mean = 9.6° ± 0.2 SE) and grasslands (mean = 7.8° ± 0.2 SE). Nevertheless, despite the significant differences between the slopes of the habitats, the difference between the mean slopes was only 9.7°.

### 3.2 Herd

Overall, the breeding herds used steeper slopes (mean = 12.6° ± 0.08 SE) than the bulls (mean = 12.0° ± 0.8 SE) (*X* ^2^ = 4.09, df = 1, *p* = 0.04).

### 3.3 Season

In contrast to our expectations, but in line with the output of the dredge function, the elephants' slope use did differ between the wet and dry seasons (*X* ^2^ = 1.21, df = 1, *p* = 0.27).

## 4 Discussion

Elephants, both African and Asian, utilise vast areas of the landscape and in doing so move across a wide range of topographical features (Nellemann et al. 2002; de Knegt 2010). Nevertheless, they generally prefer to utilise flat or gradual slopes and avoid steep portions of the landscape that are energetically costly to move along and where they are susceptible to accidents such as falling (De Knegt et al. 2011; Ngama et al. 2019; Ashiagabor and Danquah 2017). However, not all protected areas are flat. For example, a challenge for elephants in the IGR is that the reserve varies extensively with regards to topography. For these elephants, we found that 96% of their locations were on slopes < 30°, with 67% on slopes < 15°, 52% on slopes < 10°, and 15% on slopes < 5°. The use of very steep slopes was limited, with only 4% of the locations between 30° and 40°, and < 1% on slopes > 40°. Yet, the elephants in our study used steeper slopes than have been recorded elsewhere (> 15°; e.g., Wall et al. 2006; Huang et al. 2022). Factors that influenced slope use in our study included habitat and herd, with breeding herds using steeper slopes than bulls. However, the differences in slope use driving these patterns were minor.

Previous studies have suggested that elephants avoid steep slopes. For example, Huang et al. (2022) found that 95.8% of their elephant locations occurred on slopes < 3° with steep slopes (i.e., > 15°) acting as a barrier to movement by elephants in the Lower Zambezi National Park, Zambia and the Nyika National Park, Mozambique. Moreover, Ngama et al. (2029) noted that forest elephants in Monts de Cristal National Park (MCNP), Gabon, which comprises steep and variable topography similar to IGR, utilised flat (< 10% i.e., < 5°) or shallow (10%–25% i.e., 5°–15°) terrain, but avoided slopes > 15°. By contrast, only 5.8% of the elephant locations in our study occurred on slopes < 3°, while 33% were on steep slopes > 15°. In relation to the < 10° slopes preferred by Asian elephants, which are smaller and therefore presumably at less risk of severe injury after a fall (Wheatley et al. 2021), 48% of the elephant locations in our study were found on slopes steeper than this.

Despite the elephants in the IGR moving along slopes > 15°, this does not suggest that they preferred steep slopes. Rather, the elephants in our study used slopes < 30° more than expected compared to availability, and utilised slopes > 30° less than expected. When we compared our results to the categories established by Ngama et al. (2019), we found that the elephants in the IGR used the flat (> 5°) less than expected, but the shallow slopes (5°–15°) more than expected. The avoidance of flat areas may reflect heavy utilisation by other browsers and mixed feeders (e.g., kudu *Tragelaphus strepsiceros* , nyala *Tragelaphus angasii* , impala *Aepyceros melampus* ) and grazers (e.g., zebra *Equus burchellii* , wildebeest *Connochaetes taurinus* , white rhino *Ceratotherium simum* ) in the reserve, which may result in reduced food availability in these areas, but that requires further exploration. Nevertheless, our results indicate that despite the extreme topography within the IRG, the elephants still preferred to move along more gradual slopes. Yet, they did not consider slopes > 15° as a barrier.

All the habitat types, except built‐up areas, were found across the full range of slopes in the IGR. However, a distinct pattern of slope use was found to be driven by the habitat types for the elephant herds. Elephants used steeper slopes in woodlands, wooded grasslands, and bushveld compared to grasslands, riparian, and built‐up areas. However, these slopes were only marginally steeper, with the difference in the average slopes used in built‐up areas (7.8°) and woodlands (17.5°) being only 9.7°. Moreover, on average, breeding herds utilised steeper slopes (12.6°) compared to the bulls (12.0°). However, this difference is < 1°.

Biologically, the minor differences we recorded are unlikely to be meaningful. Rather, they are likely an artefact of the large sample size of elephant locations (*N* = 23,837) that we used in our analyses. Ultimately, using such a large sample allowed us to discriminate slight differences in slope use by the elephants with greater confidence (Lin et al. 2013). Despite the statistically significant results, it is unlikely that habitat and herd type play key roles in determining slope use by the elephants in the IGR. Thus, contrast to our predictions, we did not find biologically meaningful differences in slope use between the herd types, nor did season or annual rainfall provide meaning insight into their slope use. Rather, despite the undulating terrain of IGR, elephants showed a preference for more gradual slopes (i.e., < 30°) throughout the study.

A preference for flatter terrain, however, does not imply that elephants are absent from mountainous areas and steeper slopes (Rood et al. 2010; Epps et al. 2013). Lin et al. (2008) found that Asian elephants would periodically utilise slopes exceeding 50° despite these slopes being associated with a much higher risk of accidents (Wheatley et al. 2021). In our study, the steepest slope utilised by males was 53° and for breeding herds it was 58°. Looking at the 126 instances (i.e., 0.5% of the locations) where the elephants in IGR used slopes > 40°, 52% were males (*N* = 65) and 48% were breeding herds (*N* = 61), further highlighting the similar slope use by the two herd types. Seasonally, the use of steep slopes > 40° was roughly equal, with 56% of the locations occurring during the dry season (*N* = 70) and 44% (*N* = 52) in the wet season. Similar patterns are evident when exploring how the elephants utilised the 948 locations on slopes between 30° and 40°. Specifically, 53% were breeding herds (*N* = 500) and 60% were in the dry season (*N* = 559). Overall, these patterns indicate that when even focusing on just the steeper slopes, the use of these slopes was not driven by the factors that we explored. It is likely that remaining spatial autocorrelation between the model's residuals could explain and showcase insightful patterns of slope‐use by the IGR elephants, as was shown in Cushman et al. (2005) and should be explored further to gain greater insight into how elephants use slopes in parks with undulating terrain.

Despite not determining any biologically meaningful factors driving the slope use, our results allow us to estimate how much of the IGR is truly available to the elephants throughout the year. Using slopes < 30° provides an estimate of where the elephants are 96% of the time. This comes to 263 km^2^ which is 90% of the reserve. However, the elephants used slopes up to 58°. Using this as a second estimate of availability increases the total area to 291 km^2^ which is 99% of the reserve. The difference between the two categories likely represents the buffer areas that the elephants could use if food availability were to decline in areas with slopes < 30° (e.g., during droughts). However, much of the evidence suggests that elephants avoid steep slopes (Wall et al. 2006) preferring to use flat or gradual slopes. If we assume that the categories suggested by Ngama et al. (2019) and Huang et al. (2022) reflect what the elephants generally should use in IGR (i.e., they will only use slopes < 15°), then the total estimated area available to them in Ithala drops to 117 km^2^, which is only 40% of the reserve. Our data, however, show that this was not the case. As such, if 15° was used as a cut‐off when estimating the total area available to elephants in the IRG, and likely other protected areas, this would greatly underestimate the actual area they can use. This then could influence decisions on the number of elephants that a protected area could sustain. Moreover, it may underestimate the total area in which elephants could forage, and thus the impacts that they might have. This would be important if vulnerable plants were located on slopes thought to be too steep for elephants to reach, only for them to in fact be able to access those plants. Finally, with current efforts to expand the available area for elephants and linking protected areas with corridors (Douglas‐Hamilton et al. 2005; Roever et al. 2012; Pinter‐Wollman 2012; Naidoo et al. 2024), discounting areas with more undulating terrain that elephants can in fact utilise may severely limit range expansion efforts. In reserves that comprise predominantly flat slopes, the elephants' choice to avoid steep slopes and the associated risk is an obvious one. However, in a topographically complex reserves like the IGR, one should not underestimate elephants' ability to mountaineer.

## Author Contributions

**Justine M. Teixeira:** conceptualization (equal), data curation (lead), formal analysis (lead), investigation (equal), methodology (equal), writing – original draft (lead), writing – review and editing (equal). **Rickert van der Westhuizen:** conceptualization (equal), methodology (equal), writing – review and editing (equal). **Adrian M. Shrader:** conceptualization (equal), formal analysis (equal), investigation (equal), methodology (equal), project administration (lead), supervision (lead), writing – original draft (supporting), writing – review and editing (equal).

## Conflicts of Interest

The authors declare no conflicts of interest.

## Supporting information


Appendix S1.

## Data Availability

The distribution of elephant positional data poses a security risk to the animals as it exposes their preferred locations to poachers. As such, data should be requested directly from Rickert van der Westhuizen with approval dependant on the signing of a data sharing agreement with Ezemvelo KZNWildlife.
