# Supplementary material for: Despite Potential Risks African Elephants Do Not Always Avoid Mountaineering
Source: Ecol Evol. 2025 Jul 7;15(7):e71753. doi: 10.1002/ece3.71753 (PMC12234370; doi:10.1002/ece3.71753)
Supplement: Supplementary file 1 — Appendix S1. [file ECE3-15-e71753-s001.docx]

**Appendix**

**Table S1.** Functional groups for habitat that were generated from the original habitat classification by van Rooyen & van Rooyen (2008).

| **Habitat functional group** | | **Original habitat classification** | |
| --- | --- | --- | --- |
| Built up | | Built up | |
| Bushveld | | Acacia nigrescens – Combretum apiculatum rocky bushveld and woodland | |
|  | | Acacia nilotica – Acacia ataxacantha dense bushveld | |
|  | | Combretum apiculatum – Bauhinia galpinii open to dense bushveld | |
|  | | Combretum apiculatum – Diospyros lycioides subsp. nitens rocky bushveld | |
|  | | Combretum apiculatum – Euclea schimperi rocky bushveld | |
|  | | Leucosidea sericea thickets and bushveld of dolerite cliffs and scarps | |
|  | | Olea europaea subsp. africana – Euclea schimperi dense bushveld | |
| Grassland | | Cliffortia nitidula rocky montane grassland | |
|  | | Dichrostachys cinerea - Solanum incanum open disturbed patches | |
|  | | Hyparrhenia hirta - Acacia karroo old field grassland | |
|  | | Hyparrhenia hirta - Dichrostachys cinerea old field grassland | |
|  | | Hyparrhenia hirta - Sporobolus africanus old field grassland | |
|  | | Senecio microglossus - Bewsia biflora grassland | |
| Riparian | | Breonadia salicina - Ficus sycomorus riparian vegetation | |
| Wetlands | | Imperata cylindrica wetlands | |
| Wooded grassland | | Englerophytum magalismontanum - Loudetia simplex rocky wooded grassland and open bushveld | |
|  | | Greyia sutherlandii wooded grasslands of sandstone cliffs & scarps | |
|  | | Pterocarpus angolensis - Tetraselago natalensis rocky wooded grassland and open bushveld | |
|  | | Trachypogon spicatus - Themeda triandra - Euclea crispa rocky wooded grassland | |
|  | | Trachypogon spicatus - Tristachya leucothrix rocky wooded grassland | |
| Woodlands | | Faurea saligna - Searsia harveyi - Cymbopogon excavatus open woodland | |
|  | | Ficus glumosa - Euphorbia cooperi wooded rocky outcrops | |
|  | | Ficus sur - Trimeria grandifolia forests | |

**Table S2.** Adapted output for dredge function in R – with top five models, based on AIC score presented (best = 1). Variables and interactions to include indicated with +.

| **Variables** | **Models** | | | | |
| --- | --- | --- | --- | --- | --- |
|  | 1 | 2 | 3 | 4 | 5 |
| Habitat | **+** | **+** | **+** | **+** | **+** |
| Herd | **+** | **+** |  | **+** |  |
| Season |  |  |  |  | **+** |
| Year |  |  |  | **+** |  |
| Habitat x Herd |  |  |  |  |  |
| Habitat x Season |  |  |  |  |  |
| Habitat x Year |  |  |  |  |  |
| Herd x Season |  |  |  |  |  |
| Herd x Year |  |  |  |  |  |
| Season x Year |  |  |  |  |  |
| Habitat x Herd x Season |  |  |  |  |  |
| Habitat x Herd x Year |  |  |  |  |  |
| Habitat x Season x Year |  |  |  |  |  |
| Herd x Season x Year |  |  |  |  |  |
| Habitat x Herd x Season x Year |  |  |  |  |  |
| **df** | 9 | 10 | 8 | 10 | 9 |
| **AICc** | 147272.5 | 147273.2 | 147274 | 147274.3 | 147274.6 |
| **ΔAICc** | 0 | 0.65 | 1.51 | 1.76 | 2.08 |
| **weight** | 0.13 | 0.10 | 0.06 | 0.06 | 0.05 |
